# The complete chloroplast genome of *Angelica nitida*

**DOI:** 10.1080/23802359.2017.1383198

**Published:** 2017-09-29

**Authors:** Yi-Qi Deng, Jun Wen, Yan Yu, Xing-Jin He

**Affiliations:** Key Laboratory of Bio-Resources and Eco-Environment of Ministry of Education, College of Life Sciences, Sichuan University, Chengdu, P. R. China

**Keywords:** Chloroplast, genome sequence, *Angelica nitida*

## Abstract

*Angelica nitida* is an endemic species in China. The complete chloroplast genome sequence of *A. nitida* was generated by *de novo* assembly using whole genome next generation sequencing. The complete chloroplast genome was 146,512bp in length and constructed out of four parts – a large single copy (LSC) region of 93,298bp, a small single copy (SSC) region of 18,068bp and two inverted repeat (IRa and IRb) regions of 17,573bp each. The genome annotation predicted a total of 113 genes, including 80 protein-coding genes, 29 tRNA genes, and 4 rRNA genes. Phylogenetic analysis with the reported chloroplast genomes revealed that *A. nitida* is most closely related to *Angelica dahurica.*

*Angelica nitida* H. Wolff belongs to the family Apiaceae, and is a perennial herb endemic to the Qinghai-Tibet plateau region in southwest China, the growing elevation above 2400 m (Zehui and Watson 2005). The roots of this plant can replace *A. sinesis* (Oliv.) Diels as traditional Chinese medicine ‘Danggui’, and have been well used as a traditional medicinal plant for gynaecological disease, arthritis and encephalalgia (Song et al. 2014). However, *A. nitida* is not prescribed as a closely related species in the genus *Angelica* among herbal materials, mix or misuses could occur in the market place. We, therefore, assembled the complete chloroplast genome of *A. nitida*, to provide a genomic resource for fair trade and harmony in the regulation of herbal medicines through correct discrimination among the species.

The mature leaves of *A. nitida* was obtained from Jiuzhi county (33°25′51.62″N 101°28′51.41″E), Qinghai Province, China. Total genomic DNA was extracted by Plant Genomic DNA Kit. The isolated genomic DNA was manufactured to average 300bp paired-end(PE) library using the Illumina Hiseq platform, and sequenced by Illumina genome analyser (Hiseq PE150). Owing to the interference of nuclear genome, chloroplast genome related reads were sieved by mapping to the closer species *A. dahurica.* Contigs, assembled using SOAPdenovo (Luo et al. 2012), were sorted and joined into a single-draft sequence using Geneious (Kearse et al. 2012), by comparison with the chloroplast sequence of *A. dahurica* as a reference. Gapcloser was used to fill the gapped sites, and the draft sequence was corrected manually by PE read mapping using bowtie2 (Langmead and Salzberg 2012) and Tablet (Milne et al. 2013). The genes in chloroplast genome were predicted using CPGAVAS (Liu et al. 2012) and corrected by Blast search. Simple sequence repeat (SSR) motifs were investigated using NWISRL (http://ssr.nwisrl.ars.usda.gov)

The chloroplast genome of *A. nitida* (Genbank accession no. MF594405) was a circular molecular genome with a size of 14,6512bp in length, which was composed of four distinct regions such as large single copy (LSC) region of 93,298bp, small single copy (SSC) region 18,068bp and a pair of inverted repeat regions of 17,573bp.The overall GC content was 37.48%. The chloroplast genome contained 80 protein-coding genes, 29 tRNA genes and 4 rRNA genes. A total of 86 simple sequence repeats (SSRs) were discovered in the complete chloroplast genome sequence, of which tri-nucleotide SSR motif was most frequently distributed.

In order to understand the phylogenetic relationship between *A. nitida* and related species, the complete chloroplast genome sequences of 13 genera(16 species) were aligned by MAFFT (Katoh et al. 2002) and trimmed properly by trimAl (Capella-Gutierrez et al. 2009). The evolutionary history was inferred by using the Maximum Likelihood method based on Tamura-Nei model in MEGA7.0 (Kumar et al. 2016). Bootstrap (BS) values were calculated from 1000 replicate analysis. The phylogenetic tree divided into five groups, which were Apioid superclade, Acronema clade, Bupleureae, Scandiceae and Pleurospermeae (Figure 1). As was expected, *A. nitida* was closely grouped with the same genus species, and occurred as a sister taxon with *A. dahurica* with 100% BS value.

**Figure 1.**
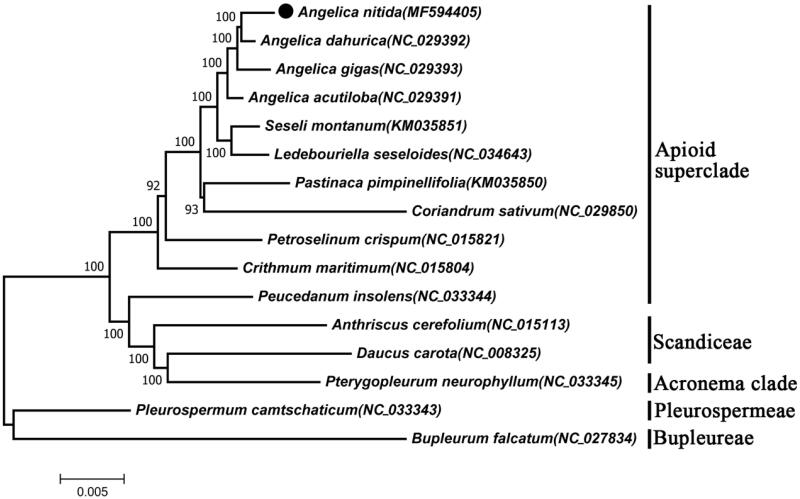
ML phylogenetic tree of *A. nitida* with 16 species was constructed by chloroplast sequence. Numbers in the nodes are bootstrap values from 1000 replicates. Bupleureae and Pleurospermeae were set as outgroup.
